# Pattern analysis for prognosis of differentiated thyroid cancer according to preoperative serum thyrotropin levels

**DOI:** 10.1038/s41598-021-01898-9

**Published:** 2021-11-16

**Authors:** Hosu Kim, Jaehoon Jung, Young-Seok Cho, Joon Young Choi, Hyunju Park, You-Bin Lee, Sun Wook Kim, Jae Hoon Chung, Tae Hyuk Kim

**Affiliations:** 1 Division of Endocrinology, Department of Medicine, Gyeongsang National University Changwon Hospital, Gyeongsang National University College of Medicine, Changwon, Republic of Korea; 2 Department of Nuclear Medicine, Samsung Medical Center, Sungkyunkwan University School of Medicine, Seoul, Republic of Korea; 3 Division of Endocrinology and Metabolism, Department of Medicine, Thyroid Center, Samsung Medical Center, Sungkyunkwan University School of Medicine, 81 Irwon-ro, Gangnam-gu, Seoul, 06351 Republic of Korea

**Keywords:** Cancer, Endocrinology

## Abstract

Serum thyrotropin (TSH) level after thyroid surgery affects the prognosis of differentiated thyroid cancer (DTC). However, the effects of preoperative serum TSH levels on the prognosis of DTC remain contradictory. In this study, to better understand the relationship between preoperative TSH levels and the prognosis of DTC, we performed pattern analysis of prognostic factors of DTC according to preoperative serum TSH levels. We retrospectively reviewed the clinical records of patients who were diagnosed and treated for DTC at the Samsung Medical Center, between 1994 and 2016. We reviewed preoperative serum TSH levels and performed a pattern analysis with prognostic risk factors for DTC. For pattern analysis, TSH was divided into 10 groups of equal fractions (TSH decile). We found a linear association between preoperative TSH levels and extra-thyroidal extension and lymph node metastasis. However, primary tumor size and initial distant metastasis showed a bimodal peak, which was similar to the pattern of overall and disease-specific death. We found that preoperative TSH range which showed the lowest mortality rate was about 0.8 to 1.59 mIU/L, which are slightly lower normal TSH levels. Although there was no linear trend, the primary tumor size, initial distant metastasis, and mortality of DTC were closely related with preoperative TSH decile and they showed a bimodal pattern. The results obtained in this study provide additional information for understanding the association between preoperative TSH levels and DTC prognosis.

## Introduction

Over the last several decades, the incidence of differentiated thyroid cancer (DTC) has been increasing worldwide^1–3^. In Korea, DTC affected approximately 43.3 per 100,000 individuals in 2014^4^. In thyroid nodules, the risk of malignancy are evaluated using preoperative ultrasonography, and the diagnosis of malignant thyroid nodules is made through a cytological examination using fine needle aspiration in most cases. Diagnostic surgery is needed for diagnosis of follicular thyroid carcinoma^5–8^. The prognosis of thyroid cancer depends on the results of pathologic findings after surgery, the result of serum thyroglobulin, and imaging test at follow up^8^. However, there are no established biochemical markers to assess the advanced thyroid cancer and poor prognosis in preoperative period.

Serum thyrotropin (TSH) is a hormone secreted by the pituitary gland and is a major growth factor for the thyroid glands. TSH regulates the development and differentiation of thyroid follicular cells via the TSH receptor. Because TSH can also stimulate the growth of thyroid cancer cells, TSH is closely associated with the risk of thyroid cancer and the stage of disease^9,10^. Recent meta-analysis showed that a positive dose response association between thyroid cancer diagnosis and serum TSH level. In this study, odds of thyroid cancer being present is 3 times greater in a patient with a serum TSH level of 4 mU/liter compared with one with a serum TSH of 0 mU/liter. In 3 of 6 studies, poor prognostic factors for thyroid cancer, such as cancer stage, tumor size, lymph node status, extrathyroidal extension, and distant metastases were also associated with increased serum TSH levels^10^. For the same reason, TSH suppression after thyroid surgery improves the prognosis of patients with DTC^8,11–13^. Therefore, serum TSH has potential to be a biochemical marker for thyroid cancer during the preoperative stage.

In a recent study, although TSH levels were within the normal range, it was reported that preoperative TSH level is associated with the advanced cancer stage^14–18^. However, the association between preoperative TSH levels and the prognosis of thyroid cancer is yet to be entirely elucidated^9,10,19,20^. This is probably due to differences in previous study designs and multiple combined variables. Therefore, in this study, we performed a TSH pattern analysis to better understand the effect of preoperative TSH on the clinicopathologic risk factors and mortality of DTC prior to surgery.

## Results

### Baseline patient population characteristics

We screened a total of 4481 patients who underwent preoperative TSH testing for thyroid surgery to treat DTC at the Samsung Medical Center between 1994 and 2016. Among them, 90 patients with preoperative TSH levels of < 0.1 mIU/L and > 10 mIU/L were excluded from the study due to suspected preexisting thyroid disease. Finally, a total of 4391 patients were enrolled and analyzed in this study. The baseline characteristics of the patients are presented in Table 1. The mean age of the enrolled patients was 46.4 ± 11.2, and 968 (22%) patients were male. Of the entire DTC patients, papillary thyroid carcinoma (PTC) accounted for 4292 (97.7%) and follicular thyroid carcinoma (FTC) 99 (2.3%). The median follow-up duration was 7.1 ± 3.3 years. One hundred forty three (3.3%) patients were relapsed during follow up period. Eighteen patients (0.4%) died during the follow-up period, and of these, 10 patients (0.2%) died due to DTC. The 5- and 10-year cancer-specific survival rates were 99.9% and 99.3%, respectively.

**Table 1:** Baseline characteristics of enrolled patients.

Characteristics	
Age at diagnosis (years)	
< 55	3398 (77.4%)
≥ 55	993 (22.6%)
Sex	
Female	3423 (78.0%)
Male	968 (22.0%)
Anti-microsomal antibody (IU/mL)	
< 60	3795 (86.4%)
≥ 60	346 (7.9%)
Thyroiditis	
No	3910 (89.0%)
Yes	481 (11.0%)
Tumor histology	
PTC	4292 (97.7%)
FTC	99 (2.3%)
Tumor size (diameter; cm)	1.04 ± 0.87
Extrathyroidal extension	
None	1995 (45.4%)
Microscopic	1866 (42.5%)
Gross	530 (12.1%)
Positive lymphatic invasion	
No	4338 (98.8%)
Yes	53 (1.2%)
Positive vascular invasion	
No	4309 (98.1%)
Yes	82 (1.9%)
Lymph node metastasis	
No LNM	2563 (58.4%)
Central LNM	1439 (32.8%)
Lateral LNM	389 (8.9%)
Distant metastasis	
No	4350 (99.1%)
Yes	41 (0.9%)
Overall deaths	18 (0.4%)
Disease-specific deaths	10 (0.2%)

Continuous data are presented as mean ± SD; categorical data are presented as absolute numbers (percentage values).

*PTC* papillary thyroid carcinoma, *FTC* follicular thyroid carcinoma, *LNM* lymphnode metastasis.

### Correlation between clinicopathologic characteristics and preoperative TSH decile

Median preoperative TSH level was 1.84 mIU/L (range, 0.1–9.41). As there was no linear increase in preoperative TSH levels, for the pattern analysis, preoperative TSH levels were grouped into equal sized decile, with each decile representing a range of serum TSH levels (Table 2). In the univariate analysis, the preoperative TSH decile was higher in older patients and patients with higher anti-microsomal antibody (AMA) levels (age: *r* = 0.031 and *P* = 0.040; AMA: *r* = 0.053, and *P* = 0.001, in the correlation analysis). The preoperative TSH decile was higher in females (5.63 ± 2.87 for females, 5.02 ± 2.82 for males, *P* < 0.001, t-test) and in patients with thyroiditis (6.03 ± 2.87 for thyroiditis, 5.43 ± 2.86 for non-thyroiditis, *P* < 0.001, t-test). In primary tumor histology, PTC had a higher preoperative TSH decile than FTC (5.51 ± 2.86 for PTC, 4.77 ± 2.73 for FTC, and *P* = 0.011). The presence of extrathyroidal extension (ETE) also showed a statistically higher preoperative TSH decile (5.74 · ± 2.88 · for presence of ETE, 5.20 ± 2.82 for no ETE, *P* < 0.001, t-test). In cases with lymph node metastasis (LNM), preoperative TSH decile was higher in patients with LNM than in those without LNM (5.62 ± 2.86, for the presence of LNM, 5.41 · ± 2.86 · for no LNM, *P* = 0.020, t-test). Preoperative TSH decile was significantly higher when lateral LNM was present (5.89 ± 2.83 · for lateral LNM, 5.46 · ± 2.86 · for no lateral LNM, *P* = 0.004). In correlation analysis, the number of LNM and preoperative TSH decile showed a positive correlation (*r* = 0.033, *P* = 0.030).

**Table 2:** Preoperative serum TSH levels decile.

	Number	Percent (%)	Median (mIU/L)	Range (mIU/L)
TSH decile 1	436	9.9	0.62	0.10–0.81
TSH decile 2	433	9.9	0.97	0.82–1.10
TSH decile 3	453	10.3	1.25	1.11–1.36
TSH decile 4	426	9.7	1.48	1.37–1.59
TSH decile 5	448	10.2	1.72	1.60–1.84
TSH decile 6	440	10.0	2.00	1.85–2.14
TSH decile 7	443	10.1	2.30	2.15–2.50
TSH decile 8	436	9.9	2.75	2.51–3.01
TSH decile 9	438	10.0	3.40	3.02–3.93
TSH decile 10	438	10.0	4.80	3.94–9.41
Total	4391	100.0	1.84	0.10–9.41

*TSH* thyrotropin.

However, the size of the primary tumor, lympho-vascular invasion, multiplicity of the primary tumor, and initial distant metastasis were not significantly correlated with the preoperative TSH decile. Recurrence also did not showed linear trend with preoperative TSH level in survival analysis (*P* = 0.527). Survival analysis for overall and cancer specific death was not performed due to a small number of events.

### Pattern analysis of DTC prognostic factor according to the preoperative TSH decile

**Figure 1:**
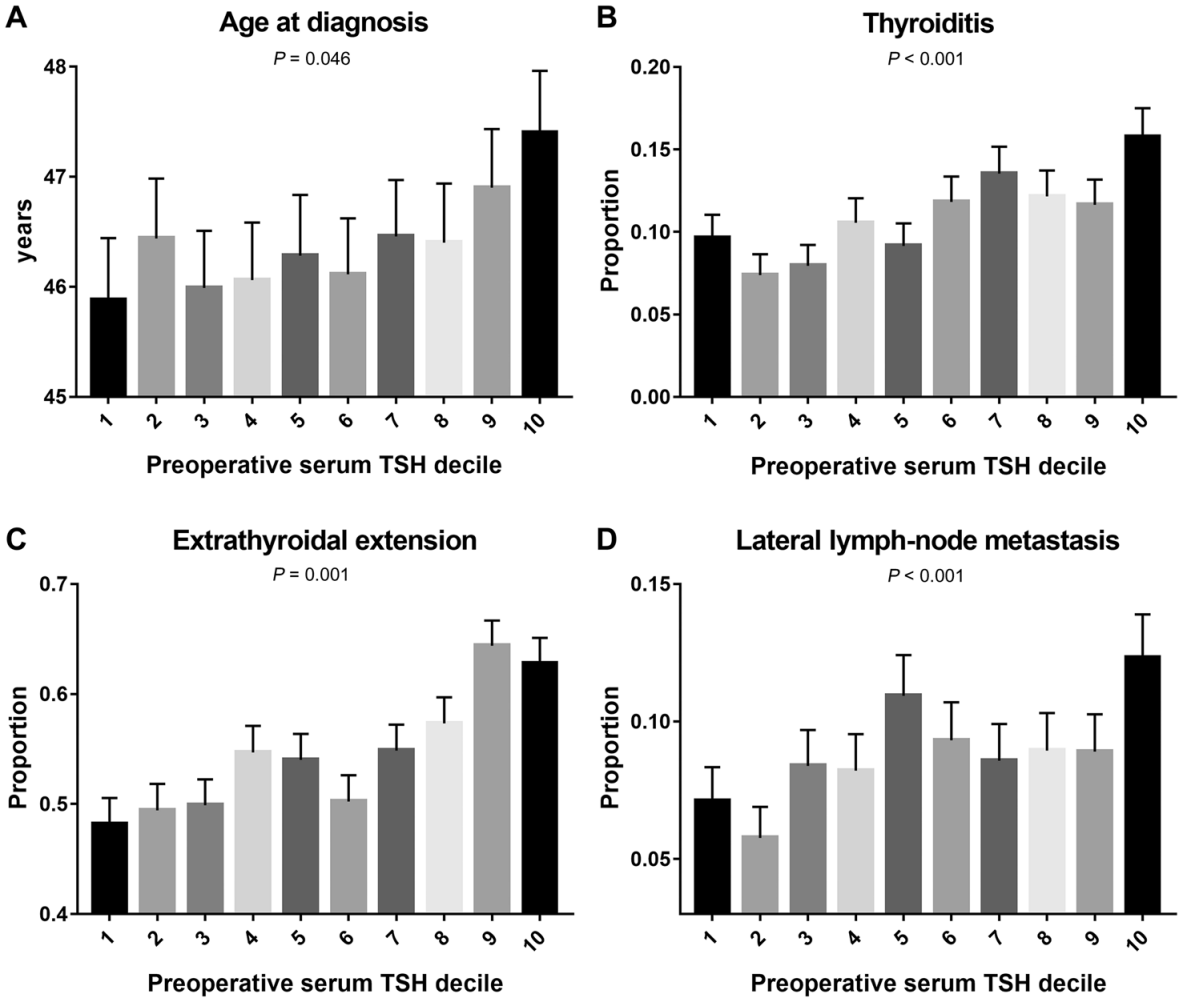
Pattern of prognostic marker of differentiated thyroid cancer according to preoperative serum TSH decile. (**A**) age, (**B**) presence of thyroiditis, (**C**) extrathyroidal extension, and (**D**) lateral lymph-node metastasis showed positive correlation with preoperative serum TSH decile.

To determine the pattern of prognosis according to the preoperative TSH decile, we calculated the ratio of the clinicopathologic risk factors and death for each group of preoperative TSH decile. In the results, similar to the results of univariate analysis, age, presence of thyroiditis, ETE, and lateral LNM were positively correlated with preoperative TSH levels (Fig. 1).

**Figure 2:**
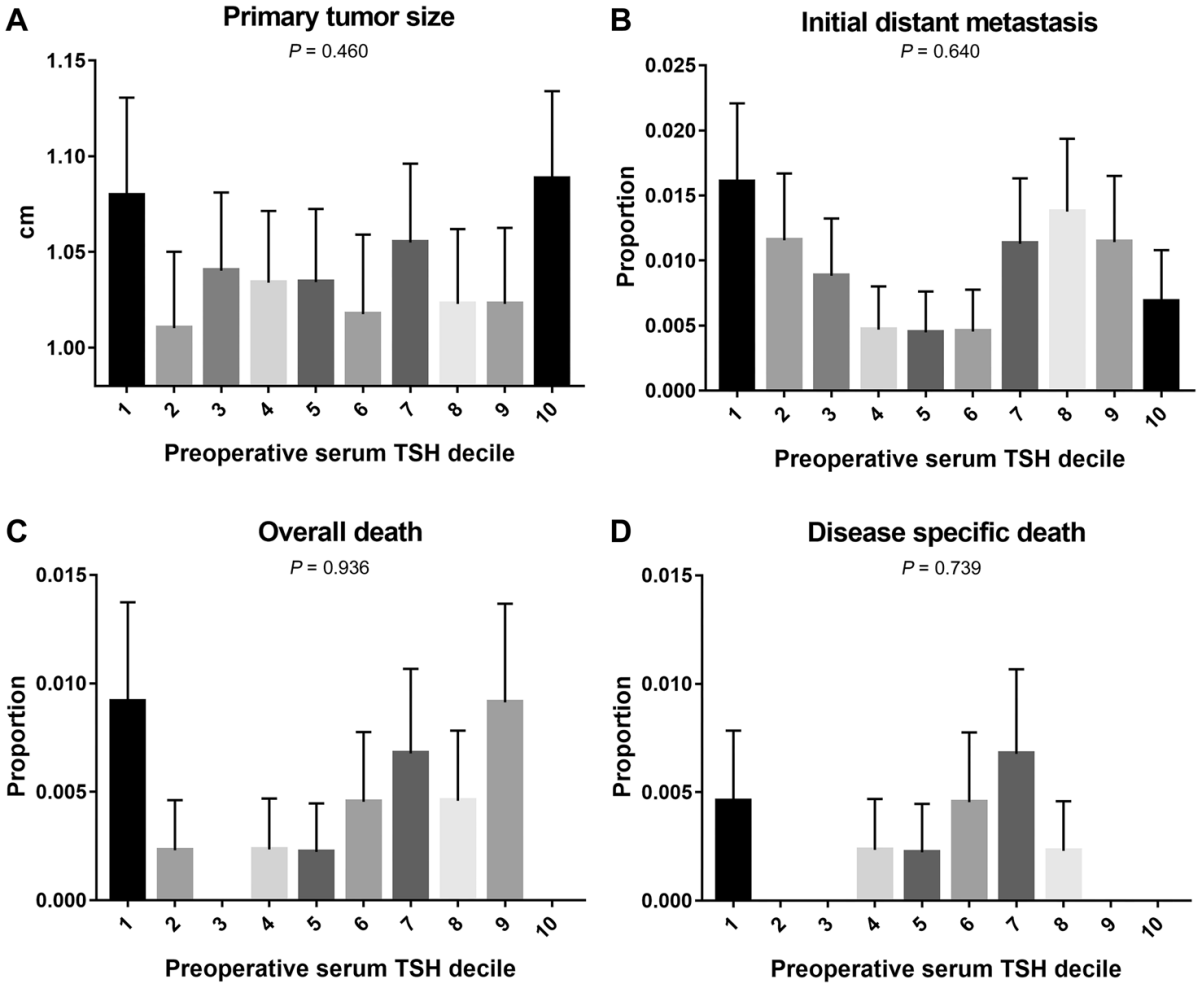
Pattern of prognostic marker and death of differentiated thyroid cancer according to preoperative serum TSH decile. (**A**) primary tumor size and (**B**) initial distant metastasis showed bimodal peak according to the preoperative TSH decile. (**C**) overall death and (**D**) disease specific death also showed bimodal peak according to the preoperative TSH decile, similar to primary tumor size and initial distant metastasis.

Specifically, primary tumor size and initial distant metastasis did not show a linear plot according to preoperative TSH decile, however, it showed a bimodal peak. Primary tumor size showed the highest fraction at preoperative TSH decile of 1 and 10 (1.08 ± 0.05 cm for TSH decile of 1, and 1.09 ± 0.05 cm for TSH decile 10). Initial distant metastasis showed the highest fraction of preoperative TSH decile 1 and 8 (7 patients for TSH decile 1, and 6 patients for TSH decile 8). Interestingly, overall death and disease-specific death also showed a bimodal peak according to preoperative TSH decile, similar to primary tumor size and initial distant metastasis. Overall death was highest at preoperative TSH decile 1 and 9 (4 patients for TSH decile 1, and 4 patients for TSH decile 9), while disease-specific death was highest at 1 and 7 (2 patients for TSH decile 1, and 3 patients for TSH decile 7) (Fig. 2).

### Subgroup analysis

As DTC prognosis varies according to age, especially age of 55, subgroup analysis was performed at the age of 55 years. In the subgroup analysis of patients under 55 years of age, the overall and disease-specific mortality rate according to preoperative TSH decile showed a bimodal peak, which was highest at preoperative TSH decile 1 and 7 (1 patients for TSH decile 1, and 2 patients for TSH decile 7 in overall and disease specific death). In the case of initial distant metastasis, preoperative TSH decile showed a high peak at 1 and 9, showing a bimodal pattern similar to mortality rate (3 patients for TSH decile 1, and 5 patients for TSH decile 9).

**Figure 3:**
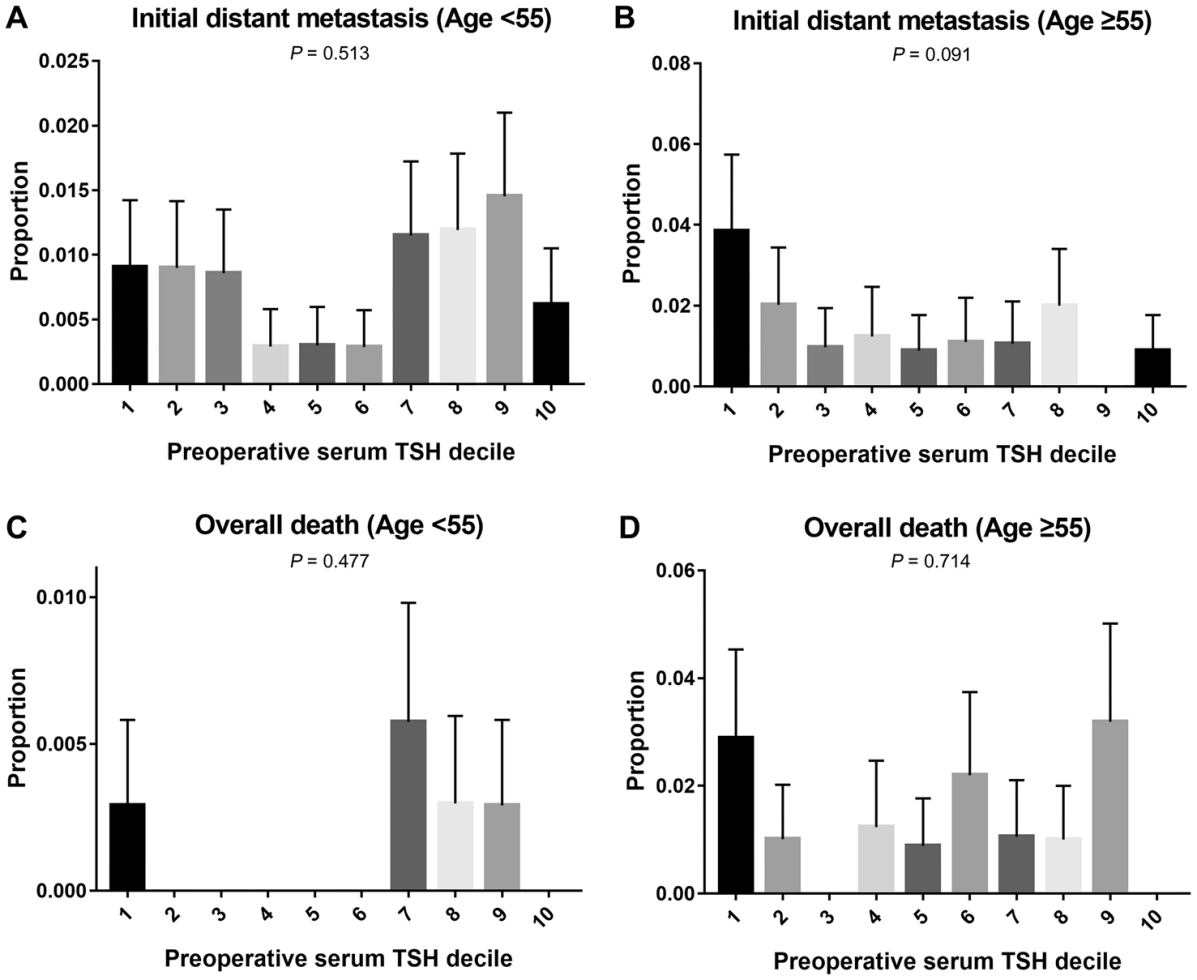
Subgroup analysis by age of initial distant metastasis and overall death according to preoperative TSH decile. (**A**) patients under 55 years of age with initial distant metastasis showed bimodal peaks. (**B**) however, negative correlation was seen in patients over 55 years of age. (**C,D**) overall death showed a still bimodal peak when subgroup analysis was performed according to age.

Regarding the subgroup analysis of patients aged over 55 years, mortality rate showed a bimodal peak, wherein overall death showed a peak at preoperative TSH decile 1 and 9 and disease-specific death showed a peak at preoperative TSH decile 1 and 6 (3 patients for TSH decile 1, and 3 patients for TSH decile 9 in overall death; 1 patients for TSH decile 1, and 2 patients for TSH decile 6 in disease specific death). On the other hand, in regard to initial distant metastasis, unlike subgroup analysis for patients under 55 years of age, it showed a negative correlation with preoperative TSH decile (Fig. 3).

The pattern of preoperative TSH decile was similar when analyzing PTC alone (excluding FTC), or excluding patients with thyroiditis, which can affect TSH (data not shown).

## Discussion

In this study, we performed a pattern analysis to evaluate the association of serum preoperative TSH levels and clinicopathologic risk factors, mortality rate of DTC. We found that preoperative TSH was positively correlated with LNM and ETE. Primary tumor size and initial distant metastasis, however, did not show a statistically significant difference in the univariate analysis. However, pattern analysis revealed a bimodal peak between the preoperative TSH decile and primary tumor size and initial distant metastasis. Furthermore, this bimodal peak is similar to the pattern of mortality rate observed according to the preoperative TSH decile.

Many studies have been previously conducted on the relationship between preoperative serum TSH levels and DTC. TSH is the main growth factor in follicular cells, regardless of the cell being normal or cancerous. However, there are no consistent results regarding the relationship between preoperative TSH levels and the prognosis of DTC. According to previous studies, preoperative TSH levels are higher in DTC than in benign thyroid nodules^10,21,22^. In addition, preoperative TSH levels are related to advanced cancer stage, and is higher in patients with LNM (particularly lateral LNM) and ETE^17,20,23,24^. However, some studies have not shown this association^25,26^. Furthermore, Medas et al. showed that lower preoperative TSH levels are associated with a high risk of thyroid malignancy, advanced tumor stage, and poor prognosis^19^. Therefore, it can be speculated that the relationship between preoperative TSH and thyroid cancer is not a simple, linear association.

Therefore, we performed a pattern analysis to clarify this relationship between preoperative TSH levels and the prognosis of DTC. As in previous studies, LNM and ETE showed a significant positive correlation with preoperative TSH levels^20,24^. Specifically, primary tumor size and initial distant metastasis, which were also prognostic factors for DTCs, were not linearly correlated with the preoperative TSH decile, but showed a bimodal peak in the pattern analysis. It is noteworthy that mortality rate such as overall death and disease-specific death also showed bimodal peaks according to the preoperative TSH decile, although the number of deceased patients was small. Therefore, it can be postulated that preoperative TSH levels are closely related to primary tumor size, initial distant metastasis, and further prognosis of DTC. This bimodal peak of prognostic factors according to the preoperative TSH decile may partially explain the contradictory results of the previous study.

Another important point in this study is the range of preoperative TSH levels that showed a lower mortality rate. Since overall death showed the lowest mortality rate at preoperative TSH decile 3 and disease-specific death at decile 2 and 3, the preoperative TSH range showing the lowest mortality rate was approximately 0.82–1.36 mIU/L. Therefore, it can be postulated that a slightly lower normal TSH level will have the best prognosis for DTC.

In our study, there were several hypotheses that may explain why the mortality rate of DTC according to preoperative TSH level shows a bimodal peak. In a patient with low TSH level, high tumor burden may exists, such as a large primary tumor size or distant metastasis^27,28^. In that occasion, TSH levels can be suppressed due to negative feedback. On the other hand, in a patients with high TSH level, increased TSH may stimulate the growth of follicular cells as well as cancer cells, resulting in local progression of tumor cells and causing clinically relevant consequences such as LNM and ETE^9,10^. Finally, it results in an advanced tumor stage and a poor prognosis.

In the subgroup analysis, initial distant metastasis did not show a bimodal peak but showed an inverse relationship according to preoperative TSH decile in patients older than 55 years. In a recent study, initial distant metastasis in elderly patients showed a high probability of telomerase reverse transcriptase (*TERT*) promoter mutations^29,30^. Therefore, through the results indicating that initial distant metastasis does not increase when TSH is high in elderly patients over 55 years of age, it can be postulated that the cancer cells of older patients are highly undifferentiated owing to the mutations, and therefore the growth stimulation caused by TSH is reduced.

There are several limitations to this study. The retrospective nature of the study and single-center design are prone to selection bias. In addition, survival time was not considered when analyzing the prognosis according to the preoperative TSH decile. Therefore, there may be some differences in survival rates. However, since most of the studies had a small number of deaths, it was difficult to directly calculate the rates. Pattern analysis may intuitively visualize the rare mortality events according to preoperative TSH level. However, the nonlinear patterns found in this study need to be replicated from independent multicenter cohorts because the number of events was very small.

## Conclusion

In conclusion, in our study, the prognosis of DTC was closely related with preoperative TSH decile and showed a bimodal pattern. Primary tumor size and initial distant metastasis were bimodal, and LNM and ETE showed a positive correlation with the preoperative TSH decile. Because TSH shows a bimodal pattern with some risk factors for thyroid cancer and mortality, researchers should be cautious when conducting research related to TSH and thyroid cancer. In addition, a slightly lower normal preoperative serum TSH level may have the best prognosis for DTC. The results obtained in this study provide additional information for understanding the association between preoperative TSH levels and DTC prognosis.

## Materials and methods

This study used an institutional thyroid cancer database. We retrospectively reviewed the clinical records of patients who were diagnosed and treated for DTC at the Samsung Medical Center between 1994 and 2016. Among them, we only enrolled patients whose preoperative TSH levels were measured. The need to obtain informed consent from the patients was explicitly waived by Institutional Review Board of the Samsung Medical Center (IRB File No. 2017-02-056) which approved this study’s protocols. This study was performed in accordance with the committee’s guidelines.

The serum TSH concentrations of patients was preoperatively checked using blood samples. Serum TSH concentrations were measured using immunoradiometric assays. The intra- and inter-assay coefficients of variation were ≤ 3.7% and 8.6%, respectively^31^. To exclude thyroid dysfunction caused by other disease such as Graves’ disease, patients with preoperative TSH concentrations of < 0.1 mIU/L or > 10 mIU/L were excluded from this study. For pattern analysis, preoperative serum TSH concentrations were divided into ten groups with equal fractions (TSH decile 1 to 10) (Table 2).

As risk factors affecting the prognosis of DTC, pathologic findings of the primary tumor, such as tumor histology, size of primary tumor (diameter, cm), lympho-vascular invasion, ETE, and LNM status were reviewed from clinical data. Regarding the status of LNM, the total number of LNM and the presence of central LNM and lateral LNM were reviewed. The presence of initial distant metastasis was ascertained from the clinical data of each patient. Initial distant metastasis was defined as a suspicious metastatic lesion observed through pathology and/or imaging (whole-body computed tomography, magnetic resonance imaging, or positron emission tomography) before surgery or within six months after surgery. Because preoperative TSH is closely related to thyroid autoimmunity, we also reviewed serum AMA levels. AMA was determined using a radioimmunoassay kit (BRAHMS AG, Hennigsdorf, Germany). When the AMA was above 60 IU/mL, it was defined as positive results to the manufacturer’s instructions. In addition, pathologically proven chronic thyroiditis was also reviewed. If there was chronic thyroiditis on the pathologic reports, it was defined as having pathologic proven chronic thyroiditis. To evaluate the patient's prognosis, first relapse, overall mortality, and disease specific mortality were reviewed. Recurrence was checked by reviewing the patient's medical record. We checked the patient's death in a national-death reporting system. The causes of death was also checked by reviewing the medical record.

### Statistical analysis

Continuous data are expressed as mean ± standard deviation, while categorical data are expressed as percentage values or absolute numbers. Non-parametric data are expressed as median and the range. For comparison of clinical and pathological characteristics according to preoperative TSH decile, Pearson and Spearman correlation analysis were used for comparison of continuous data, and the t-test or Mann–Whitney test used for categorical data. Recurrence and mortality were analyzed using the cox-proportional hazard model. Statistical significance was set at *P* < 0.05. For pattern analysis, TSH was divided into 10 groups of equal fractions. By calculating the ratio of the risk factor of thyroid cancer or death for each group, the prognosis of DTC in the TSH group was modeled. Statistical analyses were performed using the Statistical Package for the Social Sciences software version 24 (IBM Corp., Armonk, NY, USA).
